# Economic feasibility study of organic and conventional fish farming systems of Indian major carps

**DOI:** 10.1038/s41598-024-56432-4

**Published:** 2024-03-25

**Authors:** Mirza Masum Beg, Subha M. Roy, Sanjib Moulick, Basudev Mandal, Taeho Kim, Bimal C. Mal

**Affiliations:** 1 Department of Fisheries Sciences, Vidyasagar University, Midnapore, 721 102 West Bengal India; 2 Smart Aquaculture Research Centre, Chonnam National University, Yeosu, 59626 Republic of Korea; 3 School of Civil Engineering, KIIT Deemed to be University, Bhubaneswar, 751024 Odisha India; 4 Agricultural and Food Engineering Department, IIT Kharagpur, Kharagpur, 721302 West Bengal India

**Keywords:** Ecology, Zoology, Environmental sciences

## Abstract

Organic aquaculture is a new approach in the modern farming system. As the capital investment is higher for setting up the organic aquaculture, it is essential to conduct an economic feasibility study with compare the conventional farming system. In the current study, economic feasibility of culturing Indian major carps (IMC) using conventional culture system and organic culture system (OCS) were evaluated. IMC was cultured for three consecutive years from 2017 to 2019 in experimental ponds of 0.015 hectare (ha) area each. The crude protein content of the organic and conventional feed was maintained at the same iso-nitrogenous level (32% crude protein) but the highest production to the tune of 19 tons per ha was obtained in OCS. Further, in case of OCS, apart from fish production, vermicomposting to the tune of 45,000 kg ha^−1^ in the first year, and 90,000 kg ha^−1^ from second year onward is achievable by installing a vermicomposting unit of 200 tons annual capacity. Economic analysis of the culture systems assuming a project period of 10 years showed that the highest net present value (NPV) of 1.06 million USD, a payback period of one year and nine months and an internal rate of return (IRR) of 51% are achievable per ha of fish culture pond for OCS. Sensitivity analysis of various costs performed for OCS revealed that profitability of the organic fish farming investment is most sensitive to the total fish production and sale price of the organic fishes. In terms of production of fish and economics of organic culture system is proved to be the best available technique.

## Introduction

During the last few years, aquaculture made significant progress in the food sector as there is increased demand and limited supply of aquaculture products^1–5^. To meet the high demands of the food market, conventional aquaculture system has incorporated increased stocking density, usage of antibiotics, antifungal and other pharmaceuticals (mostly inorganic), heavy application of pesticides and disinfectants^6–8^ leading to environmental degradation^9–12^. These aspects demand a sustainable program through which the environment may be protected and the demands of fish protein can be met^13–16^. The sustainability of aquaculture activities is possible through organic farming^13,15,17,18^ where ecological balances of natural systems are maintained^19–21^.

The demand for the organically produced fish and fishery products is gradually increasing in the world of aquaculture^13,22–25^.The production rate of organic fisheries in the world is about 25,000 tons out of which the contribution of Europe is 14,000 tons and that of Asia and America are 8000 tons, and 3000 tons respectively^26^. For sustainable aquaculture growth in the country, a holistic approach of using natural and organic based fish feeds has to be adopted^27–30^. The input should be free from chemicals and pesticides^26,31–34^. Three Indian major carps viz. Catla (*Catla catla*, Hamilton), Rohu (*Labeo rohita*, Hamilton) and Mrigal (*Cirrhinus mrigala*, Hamilton) lead the freshwater finfish farming in India and their production has already attained commercial production level in the Indian subcontinent^35,36,56–58^. They contribute more than 70% of the total inland aquaculture production of India and more than 80% of the world production of Indian major carps.

The present study was conducted to evaluate the viability of organic fish production of Indian major carps. The Indian major carps comprising Catla, Rohu and Mrigal in the ratio of 4:3:3 were cultured in six experimental ponds following conventional culture system (CCS) (with commercial fish feed and organic and inorganic fertilizer and without aeration) and organic culture system (OCS) (with organic fertilizer and organic fish feed and without aeration). A Vermicomposting unit was established and maize and soybean crops were grown on the periphery of the fish ponds. In case of OCS, vermicomposting and liquid vermi-wash from the vermicomposting unit were utilized as organic fertilizer^37^ and the matured earthworms and organically grown maize and soybean were used as organic fish feed. Six ponds existing on the experimental farm, each measuring 0.015 ha, were used in the study. Finally, economic analysis was performed to evaluate the applicability of OCS in terms of payback period, net present value (NPV) and internal rate of return (IRR). Further, a sensitivity analysis of various costs was performed for OCS in order to determine the sensitive parameters affecting the financial aspects of the farming project.

## Materials and methods

The experiments for all animals (fishes) were conducted at Aquacultural Engineering section from June 9, 2017 to December 30, 2019, at the Indian Institute of Technology, Kharagpur, West Bengal, India, and approved by the Institutional Animal Care welfare committee and use the Ethics Committee of Indian Institute of Technology, Kharagpur, West Bengal, India with the National animal welfare guideline for animal research. This study is reported in accordance with ARRIVE (Animal Research: Reporting of in Vivo Experiments) guidelines. This study was conducted under the guidelines of the Animal Ethics Committee Regulation issued by Vidyasagar University (VU—Research Committee).

### Conventional and organic culture system

**Figure 1:**
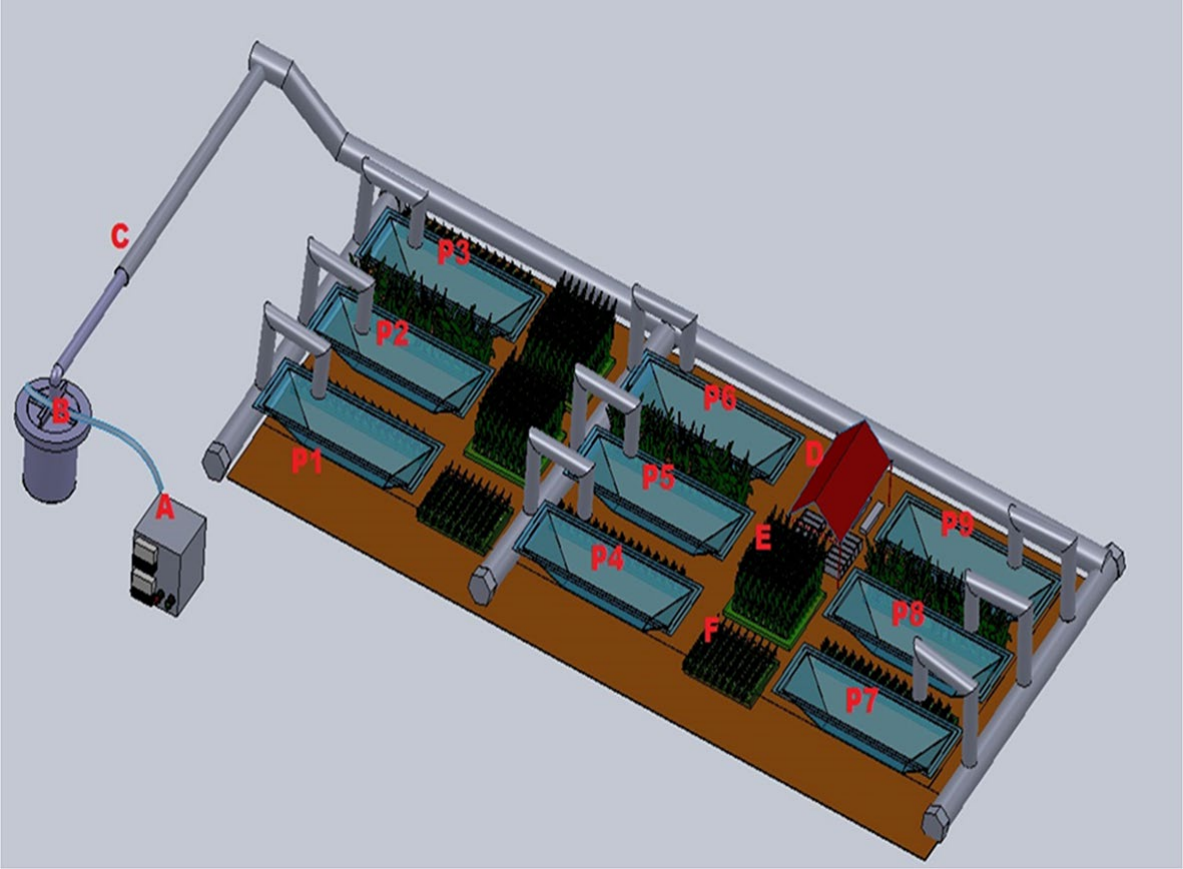
Layout of culture ponds, organic crops fields and vermicomposting unit. A - Electric facility, B - Mini deep tube well, C - Water supply pipeline, D - Vermicomposting unit, E - Maize crop field, F- Soyabean crop field, P1, P2 and P3 is the convention ponds , P4, P5 and P6is the organic ponds, P7, P8 and P9 is the reservoir ponds.

**Figure 2:**
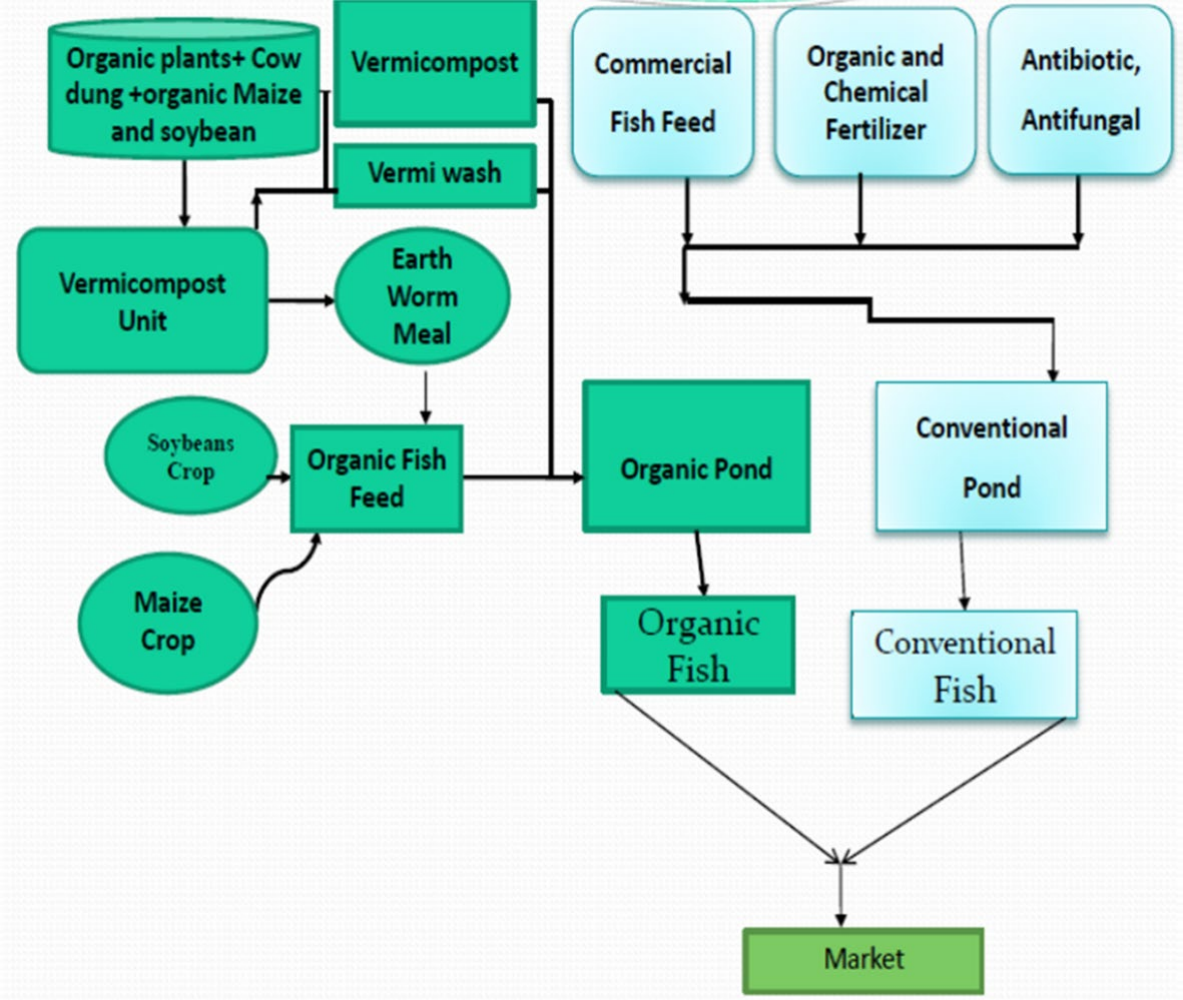
Schematic diagram showing the inputs in OCS and CCS.

In the present study, Indian major carps were cultured following two different management practices i.e., (a) conventional culture system (CCS) and (b) organic culture system (OCS). Each of the above culture practices was replicated thrice. Six ponds existing on the experimental farm, each measuring 0.015 ha, were used in the study. A schematic diagram showing the layouts of the culture ponds for CCS and OCS, organic crop fields and vermicomposting unit are presented in Fig. 1. A schematic diagram showing the various inputs used in OCS and CCS is presented in Fig. 2.

In the CCS condition, aquaculture ponds were fed with cow dung, urea and triple super phosphate (TSP) at the rate of 1250 kg ha^−1^, 31 kg ha^−1^ and 16 kg ha^−1^ respectively. The OCS condition, aquaculture ponds were fed with organic compost containing vermicomposting and vermin bed-wash at the rate of 12,443 kg ha^−1^ and 93.33 L ha^−1^ respectively^38^. Vermicomposting as well as vermi bed-wash were distributed uniformly over the water surface of the ponds^39^.

The fingerlings of three Indian major carps, Catla, (*Catla catla,* Hamilton), Rohu (*Labeo rohita*, Hamilton) and Mrigal (*Cirrhinus mrigala*, Hamilton) were stocked with a ratio of 4:3:3 at the rate of 10,000 fingerlings ha^−1^^44^. Good quality and disease free equal size fingerlings were stocked for better growth rate^34,40,53–55^. The fish fingerlings were free from genetically modified organism (GMO) and genetic engineering^22,41,42^. The certified seed was collected from local hatchery, Kharagpur, West Bengal, India. Stockings were done in the early mornings, usually before 9.30 AM when the temperature of water was low. Before stocking, the fish fingerlings were kept in a 2% NaCl solution bath for 1–2 min and were well acclimatized to pond conditions. The mean initial weights of IMC stocked were: Catla: 25.5 ± 1.09 g, Rohu: 22.5 ± 1.08 g and Mrigal: 21.3 ± 1.06 g. In CCS, commercial feed and antibiotics were used. The commercial fish feed was pelleted feed (2–4 mm diameter) produced by a local commercial fish feed company (CPF Pvt. Ltd, India). In OCS, earthworm and organically grown protein and oil rich crops, soybean and maize were used as ingredients of organic pelleted fish feed. The crude protein content of the organic and conventional feed was maintained at iso-nitrogenous level (32% crude protein). Pelleted feed was fed twice a day in both conventional and organic ponds. The pelleted feed was provided at the rate of 5% of fish biomass up to 30 days, 3% up to 60 days, 2% up to 160 days, and 1% up to the rest of the culture period^7^. Survival rate was both culture patterns 90%.

The harvesting of fishes was done through the repeated netting and draining of ponds at the end of nine months of the culture period. The survival rates and various yield parameters of fishes were recorded during the experiments. The sale price of fishes was primarily dependent on wet weight at the time of harvesting and type of culture provided^43^. The organically grown fish fetched approximately 30% higher price (premium price) than that of conventionally grown fish. Sale price of conventionally grown fish (non-organic) was USD 2.17 kg^−1^ in the local fish market, Kharagpur, West Bengal, India, the average weight being above 600 g (Catla: 630 ± 5.6 g, Rohu: 670 ± 5.5 g and Mrigal: 504 ± 5.3 g) and that for the organically grown fish was USD 2.75 kg^−1^, where average fish weight was above 700 g (Catla 709.5 ± 4.3 g, Rohu 708.4 ± 4.2 g and Mrigal 547.7 ± 4.2 g). The proximate composition of earthworm and formulated fish feeds were estimated following AOAC (2003) method. Crude protein was determined by macro-Kjeldahl method using Kjeldahl Apparatus (BUCHI). Ash content was found out by weighing the sample after it was subjected to 500 °C in a muffle furnace. Determination of ether extract or crude fat was done by ether extraction method. Crude fiber was found out by acid digestion of residues from the ether extraction and loss in weight on ignition. Gross energy was evaluated using bomb calorimeter (Parr 6300 Calorimeter, Moline, IL, USA), with benzoic acid as a standard. Proximate composition of fish feed. The ingredients, proximate composition and energy content of conventional and organic feed are presented in Table 1.

**Table 1:** Ingredients, proximate composition and energy content of conventional and organic feed.

Ingredients	Conventional feed	Organic feed (Organically grown all Ingredients)
Fish meal	25.60	–
Soybean Meal	35	39
Mustard oil cake	24.4	–
Wheat flour	13.0	–
Vitamin & minerals	2.0	2.0
Earthworm meal	–	18.5
Maize meal	–	32
Coconut cake	–	8.5
Proximate composition		
Protein	32 ± 0.3	32 ± 0.4
Carbohydrate	38.11 ± 0.7	35.39 ± 0.5
Fat	7.39 ± 0.8	9.40 ± 0.4
Ash	13.34 ± 0.5	13.96 ± 0.4
Moisture	9.16 ± 0.6	9.25 ± 0.9
Energy contents (kJ g^−1^)	14.76 ± 0.1	14.93 ± 0.3

### Determination of fish growth parameters

More than 30% of the fish of all the tanks were sampled fortnightly and individual measurements were taken to determine the fish yield parameters. Growth performance was examined using specific growth rate (SGR), feed utilization and net weight gain determination by feed conversion ratio (FCR).The two major fish growth parameters are (i) specific growth rate, and (ii) feed conversion ratio (FCR). These parameters were calculated using the following equations:

1 $${\text{SGR}}\left( {\% \;{\text{body}}\;{\text{weight}}/{\text{day}}} \right) = 100 \, \times \frac{{\ln \left( {Final\;weight - Initial\;weight} \right)}}{{Culture\;period \left( {days} \right)}}$$

2 $${\text{FCR}} = \frac{{Amount\;of\;feed\;eaten \left( {dry\;weight\;basis} \right)}}{{Net\;weight\;gain \left( {wet\;weight\;basis} \right)}}$$

### Statistical analysis

Data obtained on different proximate composition of feed and fish growth parameters were analyzed by one-way ANOVA with different culture systems (CCS and OCS) as the factor. Post-hoc comparisons were made using Duncan’s new multiple range test to detail the significant differences among the treatments (*P* < 0.05). All statistical analyses were performed using SPSS version 17.

### Economic analysis

The economic analysis included determination of expenditure and income; profit; payback period; net present value and internal rate of return. The returns on such small pond area (0.15 ha) are very less and at times can be negative also. Therefore, to compare the economics of different alternatives 1.0 ha pond area was considered. The cost of various items was suitably scaled up for 1.0 ha area based on the cost involved in 0.015 ha area. In fact, average weight of fish with the same stocking density and under the same management practice is expected to be more in relatively bigger sized ponds with the same depth. In bigger sized ponds, fishes can traverse a greater distance and therefore, exercise more leading to better growth^44^. Therefore, the analysis made based on the yield of smaller ponds is on the safer side. In case of OCS, the area required for vermicomposting unit and organically grown maize and soybean crop were also suitably scaled up to meet the demand for 1.0 ha organic fish pond. The profit, payback period, net present value (NPV), and internal rate of return (IRR) were calculated for CCS and OCS using the following formulae:

3 $${\text{Profit}} = {\text{Income}}{-}{\text{operating}}\;{\text{cost}}$$

4 $${\text{Payback}}\;{\text{period}} = {\text{Initial}}\;{\text{outlay}}\;\left( {{\text{IO}}} \right)/{\text{cash}}\;{\text{flow}}$$

5 $${\text{NPV}} = \mathop \sum \limits_{t = 0}^{n} \frac{{CF_{t} }}{{\left( {1 + k} \right)^{t} }} - IO$$

where CF is the cash flow over the time of the project; IO is the initial outlay; k is the discount rate of bank interest rate with a value of 10%, t is the time period and n is the life time of the project.

Internal rate of return (IRR) was calculated by determining the value of the discount rate at which NPV becomes zero.

The cost analysis of CCS and OCS includes two types of costs: (a) Initial investment cost for creation of facility for culturing of fishes and (b) the variable costs involving the maintenance costs of the fishpond, land lease cost, cost of fingerlings, cost of feed, fertilizer and production cost of field crops.

- Initial investment

The initial investment includes mainly the cost of pond construction, water facilities and vermicomposting unit. Earthwork for construction of the ponds was carried out by engaging a contractor who executed the work by engaging daily laborers. The work was carried out according to the requirement of the site. As the soil cannot retain water, it was felt necessary to use lining material on the excavated ponds. Therefore, steps were made from ground level to the bottom of the fishpond for better anchoring of the lining material. Prismoidal formula was used to compute the volume of earthwork. The cost of earthwork was paid to the contractor as per the schedule of rates 2017 of the Public Works Department (PWD), Government of West Bengal, India. As per the schedule, the cost of earthwork for first 1.5 m depth (lift) was USD 0.29 m^−3^ and USD 0.40 m^−3^ for the next 1.5 m depth. The price of lining includes the cost of lining material and labor wage to spread and bury it with soil. Cross laminated polythene sheet of Sylpaulin make (250 µ thick, 150 g m^−2^ weight, UV ray protectable and green in color) was used for lining of the dugout fishponds. The actual price of the Sylpaulin (a plastic film for coverings) sheet charged by the authorized dealer was considered to compute the cost of lining material. The price was USD 0.51 m^−2^ in the year 2017. Labor wage required to spread the polythene sheet on the bottom and sides, including the embankment and to bury the same with a soil layer of 30 cm thickness was paid as per the schedule of rate of Government of West Bengal, India, 2017. Before laying the cross laminated polythene Sylpaulin material on the bed of the fish ponds, a sand cushioning was provided to a depth of 20 cm to avoid any rupture. After laying the Sylpaulin material, a soil cover of 30 cm thickness was also provided on the lining material to provide stability to the material and create a natural pond bottom environment for fish culture. The number of laborers required for the job was 5 man per days for sand filling, earth filling and providing lining material in one pond. A mini deep tube well along with underground pipe lines and accessories was constructed for regular water supply to the fish ponds. The cost for vermicomposting unit included the maintenance costs of the land and building, civil works for vermicomposting shed and vermicomposting tanks, implements and machinery and others work.

(b) Variable costs

The variable costs included maintenance costs of the fishpond, land lease cost, cost of fingerlings, cost of feed, fertilizer and production cost of field crops. The Maintenance cost of the fishpond involves the expenditure incurred for repair and maintenance of the embankment. The cost has been assumed to be 2% of the initial investment^1,45^. It was thought appropriate to add the annual land lease cost for the area diverted for the construction of fishpond. The cost was decided based on prevailing lease rate under the revenue district of West Medinipore, West Bengal, India. The cost was found to be USD 60.71 ha^−1^ year^−1^ as per the rate of 2017. Fingerlings were purchased from a nearby farm for stocking in the fishponds. The cost of fingerlings varies depending on the size, weight etc. Fingerlings were purchased at USD 2.17 kg^−1^ and later released to the fishponds after acclimatizing them in an earthen tank for 40 h. The organic fish feed was prepared in the laboratory (Aquacultural Engineering Lab, IIT Kharagpur, West Bengal, India) with due proximate composition of suitable protein, carbohydrate, fat, ash, etc. Conventional feeds were bought from the local feed company and the formulated fish feed cost was only USD 0.51 kg^−1^ at the prevailing cost of inputs in 2017. The chemical fertilizer cost was calculated as per the local market.

### Ethical statement and consent to participate

The study was approved by the Ethics Committee of “IIT Kharagpur”, West Bengal, India. All experiments were performed in accordance with ARRIVE guidelines (PLoS Bio8 (6), e1000412, 2010). This study was conducted under the guidelines of the Animal Ethics Committee Regulation issued by Vidyasagar University (VU –Research Committee).

## Results and discussion

### Growth performance

A one-way ANOVA was performed to compare the effects of initial, final weights, specific growth rate (SGR) and FCR (Feed conversion ratio) of Catla, Rohu and Mrigal on CCS and OCS system are presented in Table 2.

**Table 2:** Analysis of variance (ANOVA) for CCS and OCS.

Source of variation	SS	DF	MS	F	*P* value
Initial and final weight CCS					
Between groups	10,310,812	5	20.62	13.13	0.03
Within groups	1792.925	114	15.72		
Total	10,312,605	119			
Initial and final weight OCS					
Between groups	12,334,609	5	24.66	25.66	0.02
Within groups	1095.929	114	9.61		
Total	12,335,705	119			
SGR CCS					
Between groups	0.099	2	0.049	0.066	0.03
Within groups	42.85	57	0.751		
Total	42.95	59			
SGR OCS					
Between groups	0.063	2	0.031	0.019	0.03
Within groups	91.603	57	1.607		
Total	91.666	59			
FCR CCS					
Between groups	0.016	2	0.008	12.168	0.04
Within groups	0.037	57	0.0006		
Total	0.054	59			
FCR OCS					
Between groups	0.007	2	0.003	109.592	0.02
Within groups	0.001	57	3.29E−05		
Total	0.009	59			

A one-way ANOVA of initial weights of CCS and OCS were identical. The final weights of CCS and OCS revealed that there were a statistically significant difference in mean CCS and OCS systems (F = 13.13 and 25.66 and *P* < 0.05). The SGR of CCS revealed that there was a statistically significant difference in mean CCS systems (F = 0.066 and *P* < 0.05). The SGR of OCS revealed that there was a statistically significant difference in mean at least two groups OCS systems (F = 0.0197 and *P* < 0.05). The FCR of CCS revealed that there was a statistically significant difference in mean CCS systems (F = 12.16 and *P* < 0.05). The FCR of OCS revealed that there was a statistically significant difference in mean OCS systems between (F = 109.57 and *P* < 0.05).

The initial stocking weight (g) and final harvested weight (g) of Indian major carps for both the culture systems are presented in Table 3. It can be seen from the table that the organic culture system contributed greater individual weight gain and net fish production than the CCS. It is due to the fact that good quality organic feed was used in the OCS system and it is favorable for better growth of fishes. Organic fishes are known to grow better in more protected conditions than conventional fish^45,46,52,53^. Fishes stocked in the experimental ponds had almost the same mean initial weight (Catla: 25.5 ± 1.09 g, Rohu: 22.5 ± 1.08 g and Mrigal: 21.3 ± 1.06 g) for both treatments without any significant variation (*P* < 0.05). Individual harvested size was higher in OCS culture (Catla 709.5 ± 4.3 g, Rohu 708.4 ± 4.2 g and Mrigal 547.7 ± 4.2 g), compared to the CCS (Catla: 630 ± 5.6 g, Rohu: 670 ± 5.5 g and Mrigal: 504 ± 5.3 g) culture system.

**Table 3:** Mean values (Mean ± SD) of the initial and final weight of fish with specific growth rate and feed conversion ratio in CCS and OCS.

Fish Species	CCS						OCS					
	Weight (g) (Mean ± SD)		SGR (specific growth rate (%day^−1^))		FCR		Weight (g) (Mean ± SD)		SGR (specific growth rate (% day^−1^))		FCR	
	Initial weight	Final weight	Mean ± SD	Range	Mean ± SD	Range	Initial weight	Final weight	Mean ± SD	Range	Mean ± SD	Range
Catla	25.5 ± 1.09^a^	629.7 ± 5.66^b^	1.190 ± 0.81^a^	0.041–2.59	1.64 ± 0.01^a^	1.61–1.66	25.5 ± 1.09^a^	709.55 ± 4.32^c^	1.235 ± 0.86^b^	0.334–2.899	1.38 ± 0.01^a^	1.36–1.38
Rohu	22.5 ± 1.08^b^	669.7 ± 5.52^c^	1.279 ± 0.92^b^	0.328–2.875	1.60 ± 0.03^c^	1.60–1.63	22.5 ± 1.08^b^	708.41 ± 4.23^a^	1.298 ± 0.91^c^	0.185–2.544	1.36 ± 0.01^b^	1.36–1.39
Mrigal	21.3 ± 1.06^a^	503.73 ± 5.32^b^	1.194 ± 1.01^b^	0.121–3.284	1.62 ± 0.03^b^	1.62–1.65	21.3 ± 1.06^a^	547.73 ± 4.21^b^	1.224 ± 1.80^c^	0.012–3.724	1.39 ± 0.01^c^	1.36–1.35

^a,b,c^Means superscripted with different letters in the same row are significantly different (*P* < 0.05).

The growth performance indicator, specific growth rate (SGR) of Indian major carps in different culture systems is presented in Table 3. Post-hoc comparisons were made using Duncan’s new multiple range test to detail the significant differences among the treatments (*P* < 0.05).The peak value of SGR (1.29% day^−1^) was recorded in Rohu in OCS. The SGR values of all fishes in OCS were significantly different (*P* < 0.05) from other culture systems with no negative effects on growth^6,45,47^. The growth performance and feed utilization (SGR and FCR) during the experiment were different in the two culture systems suggesting that the organic diet did not induce chronic stress, with long-term detrimental effects on growth^17,48^. It is corroborated by the same results found in European sea bass^49,51^ multi species combination culture in rice fish system^5^. It is seen from Table 3 that non-significant variation of FCR was recorded between conventional culture system and organic culture systems (*P* < 0.05). The maximum FCR value was recorded in CCS (1.64) and the least value of FCR was recorded in OCS (1.38).

### Economics analysis

#### Expenditures and income

The common items required in two management systems, i.e., CCS and OCS are soil excavation, polythene sheet, sand and bricks and labor charges for miscellaneous works. For excavation of 1.0 ha pond with a step close to the middle of the slope of the embankment, approximately 14,300 m^3^ soil needs to be excavated for a maximum depth of 1.5 m at the center of the pond. At the rate of USD 0.25 per m^3^ of soil excavation, a sum of USD 3613.88 was required for excavation of the pond. The rate of the polythene sheet is USD 0.49 per m^2^. For, 1.0 ha pond lining the requirement of sheet is about 10,400 m^2^. So, the total cost of polythene sheet for pond lining is around USD 5111.46. Before polythene lining, a 20 cm layer of sand is needed to be spread on the pond bottom to provide cushioning effect to the sheet. For this 2000 m^3^ of sand costing USD 2023.77 is needed. To prevent sliding of the polythene sheet from the embankment, bricks are to be placed at regular intervals on the steps over the sheet. If the bricks are placed on two steps continuously lengthwise on a 100 m × 100 m pond, about 3200 bricks costing USD 231.29 are needed. Construction of polythene lined pond is more labor intensive compared to that of a natural pond. Labor is required for preparation of sand bed, softening of the slope of the embankment with water and sand, sieving of sand and spreading on the pond bottom, laying of the polythene sheet on the pond bottom by joining it as per required length, powdering, cleaning and putting the soil on the bottom, putting the bricks and soil on the step of the embankment etc. For all these purposes, an estimated 500 man days with a total expenditure of USD 578.22 are needed. A sum of USD 289.11 is allotted for miscellaneous expenditures.

The total capital expenditure on different heads is presented in Table 4. The recurring expenditure is found to be more in organic culture system compared to the conventional culture systems as shown in Table 5.

**Table 4:** Initial investment (USD) for construction of one ha farm for CCS and OCS.

Sl. no	Particulars	CCS	OCS
1	Cost of Pond Construction		
i	Soil excavation	3613.88	3613.88
ii	Polythene sheet	5111.46	5111.46
iii	Brick & Sand	578.22	578.22
iv	Labor or different works	578.22	578.22
v	Miscellaneous expenditure	2911.34	289.11
2	Water facilities (Tube well with water supply facilities, pumps etc.)	1373.27	1373.27
3	Vermicomposting unit	–	17105.19
Total		14166.39	28649.35

**Table 5:** Capital cost of vermicomposting unit.

Sl. no	Particulars of item	Amount (USD)
A.	Land and Building	
1.	Land (on rent/lease)	–
2.	Levelling and soil filling for vermicomposting sheds	343.32
3.	Fencing and gate	596.29
4.	Open shed with brick lined bed bottom & platform with RCC/MS pipe post & truss and thatched/HDPE locally available roof (@USD14.46/m^2^) for:	
(a)	vermicomposting beds (15 m × 1.5 m × 24 (nos.) = 540 m^2^ + 20 m^2^ pathways/utility = 560 m^2^)	8095.08
(b)	For finished products 30 m^2^	433.66
5.	Godown/Store cum office 50 m^2^ @ USD72.28/-per m^2^	3613.88
Sub total		12612.42
B.	Implements and machinery	
1	Shovels, spades, crowbars, iron baskets, dung fork, buckets, bamboo baskets, trowel	72.28
2	Plumbing and fitting tools	21.68
3	Power operated shredder	361.39
4	Sieving apparatus with 3 wire mesh sieves—0.6 m × 0.9 m size—power operated with motor	650.50
5	Weighing scale (100 kg capacity)	36.14
6	Weighing machine (platform type)	86.73
7	Bag sealing machine	72.28
8	Culture trays (35 cm × 45 cm)—4 Nos	23.13
9	Wheel barrows—2 Nos	173.47
Subtotal		1497.59
C.	Water provision—Borewell with hand pump, pipe, dripper	1084.16
D.	Electrical installation	144.56
E.	Furniture & fixtures	361.39
F.	Earthworms (@1 kg per m^3^ and @ 300/kg, total utilized bed volume = 324 m^3^)	1405.07
Total capital cost		17105.19

### Expenditure of vermicomposting unit

**Figure 3:**
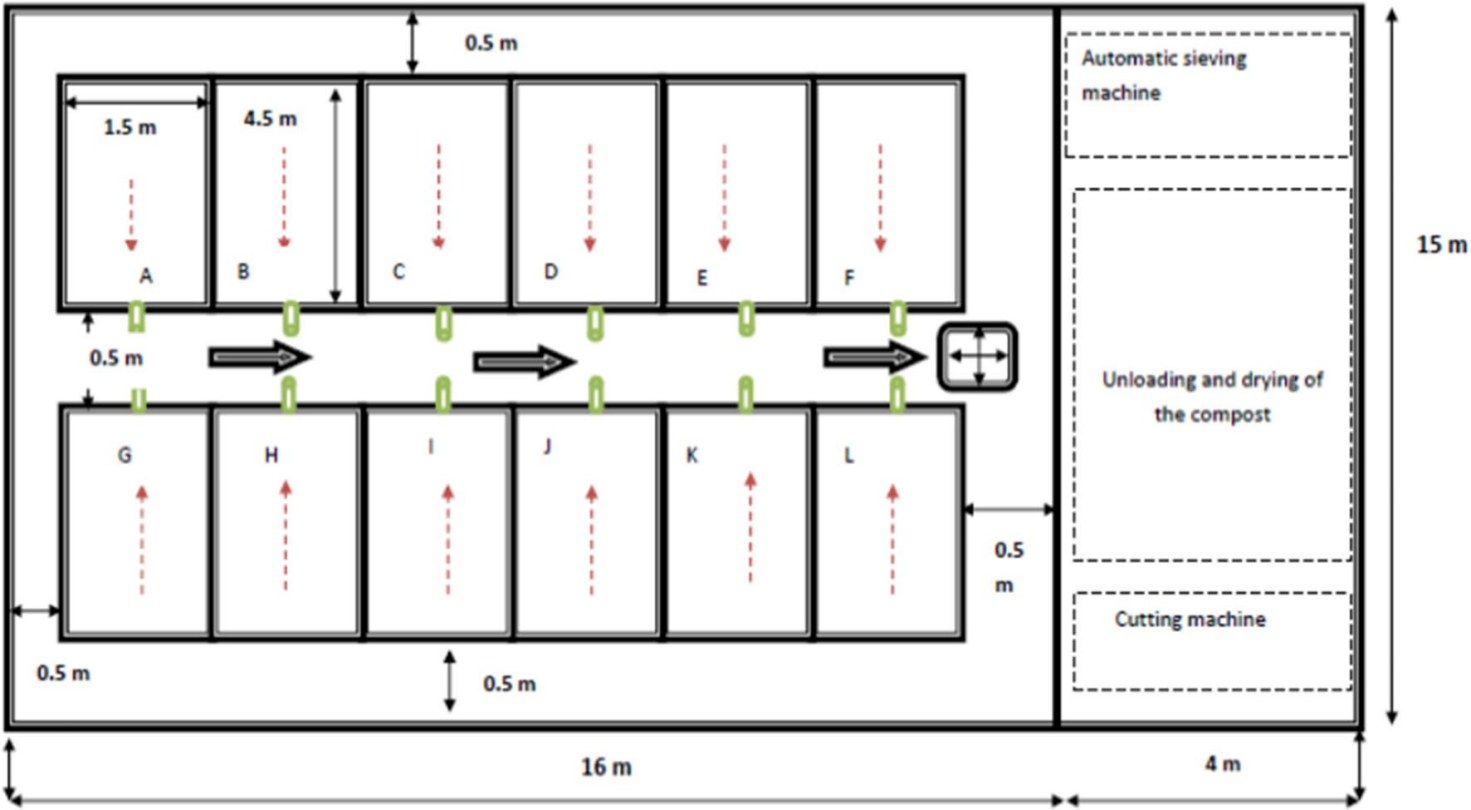
Design of vermicomposting unit comprising 12 nos. of Vermicomposting beds (4.5 m × 1.5 m × 0.5 m) and arrangements for automatic sieving, unloading and drying of compost and chopping machine.

The organic culture system, vermicomposting unit with a capacity of 200 tons per annum (TPA) was developed as shown in Fig. 3. The capital cost of vermicomposting unit (200 TPA) is presented in Table 5. National Bank of Agriculture and Rural Development (NABARD, 2014). The operational cost and the cost-benefits are shown in Tables 6 and 7, respectively. The cost of cultivation and return from soybean were evaluated for all the three experimental seasons. The investment cost incurred for the cultivation of soybean crop is listed in Table 8. The average cost for soybean cultivation was USD 350.18 ha^−1^. The farm produced 1.0 ton of soybean and selling price was USD 578.22 per ton during the experimental season. About 5% of the yield is assumed to be spoiled and a net return from 95% of soybean yield was taken as the net income. The net return from the soybean cultivation is presented in Table 9.

**Table 6:** Operational costs of vermicomposting unit of 200 tons per annum (TPA).

Sl. no	Particulars of item	Year 1 Amount (USD)	Year 2 Amount (USD)
1	Agricultural wastes (cost, collection and transportation) @ 320 kg per m^3^ and USD 2.90/MT (15 × 1.5 × 0.6 × 24 × 5 × 320 × 200/1000) [at 50% in 1st year]	374.70	749.3
2	Cow dung (cost, collection and transportation) @ 80 kg/m^3^and USD 3.61/MT(15 × 1.5 × 0.6 × 24 × 5 × 80 × 250/1000) [at 50% in 1st year]	234.18	468.36
3	Salary pay for 2 stable skilled laborers @ USD 86.73/month	173.47	173.47
4	Labor pay on day to day basis in development of vermin bed with agro-waste, cow dung and worms, watering, stirring, harvesting, sieving, packing, etc., including cost of bags. [at 50% in 1st year]	361.39	722.77
5	Electrical charges for pump, machinery, lighting etc. [at 50% in 1st year]	173.47	346.93
6	Repair and maintenance [at 50% in 1st year]	433.66	867.33
7	Cost of bags and marketing cost [at 50% in 1st year]	216.83	433.66
Sub total		1967.69	3414.96
8	Lease rent, Miscellaneous etc	346.92	780.48
Total operational cost		2314.61	4195.56

**Table 7:** Cost and benefits of vermicomposting unit’s 200 tons per annum (TPA).

Sl. no.	Cost and benefit	Amount (USD)	
		Year 1	Year 2 onwards
1.	Total capital cost	17105.19	–
2.	Total operational cost	2314.61	4195.56
3.	Total cost	19419.81	4195.56
4.	Benefit		
4a.	Sale of vermicomposting (200 MT @ 30% conversion) [@USD 65.05/MT at 60% in 1st year and 90% in 2nd year onwards]	2927.24	5854.48
4b.	Total benefit	2927.24	5854.48

**Table 8:** Average cost of cultivation for soybean and maize.

Sl. no.	Input parameters	Soybean cultivation cost per hectare (USD ha^−1^)	Maize cultivation cost per hectare (USD ha^−1^)
1.	Land lease cost	60.71	60.7
2.	Land preparation (LS*)	72.28	57.2
3.	Fertilizer	–	–
4.	Seed	43.73	2.0
5.	Plant protection (LS)	28.91	14.5
6.	Labor (man-days)	144.56	144.6
7.	Total cost of cultivation	350.18	279

*LS, Lump sum.

**Table 9:** Net return (USD ha^−1^) from soybean and maize cultivation.

Treatment	Year	Net yield (t)	Average cost (USD ha^−1^)	Net return (USD ha^−1^)	Average net return (USD ha^−1^)
Organically grown soybean	2017	1.02	350.18	210.11	–
	2018	1.48	-do-	462.79	323.63
	2019	1.18	-do-	298.00	–
Organically grown maize	2017	3.705	278.99	150.77	–
	2018	3.90	-do-	172.02	151.20
	2019	3.63	-do-	140.80	–

The average cost for maize cultivation is USD 279 ha^−1^. The farm produced 3.0 tons of maize and selling price was USD 151.20 per ton during the experimental season. The investment cost for cultivation and the net return obtained from sale of maize are listed in Tables 8 and 9, respectively.

### Economic indicators

#### Profit

The details of input costs in terms of seed, feed, lime, fertilizers, netting, for water exchange, labor and prophylactics during culture period and the income generated by selling the fishes are presented in Table 10. The income from selling of fish for conventional culture system is USD 13094.63. However, in organic culture system, an income of USD 19770.10 in first year and USD 22046.86 from second year onwards can be generated from selling of fish. The maximum selling price of fish is obtained in the organic culture system (USD 2.75 kg^−1^) due to their better size and consumer acceptability; whereas the selling price of fish is USD 2.17 kg^−1^ for CCS. The profit (32,328 USD) of the Organic culture system (OCS) mentioned in Table 11 is considering first two years of culture, while that for Conventional culture system (CCS), only 1st year was considered.

**Table 10:** Input costs (USD) and income for conventional and organic culture system.

Items	CCS (USD)	OCS (USD)	
		1st year	2nd year onwards
Input costs			
Lime	32.50	35.78	2475
Cow dung	21.70	–	–
Urea	2.60	–	–
SSP	2.31	–	–
Fish fingerlings	289	289	289
Feed	4337	–	–
Water filling	43.4	43.4	43.4
Netting	87	87	87
Prophylactics	43	–	–
Labor	289	361	361
Soybean crop	–	350	350
Maize crop	–	279	279
Vermicomposting unit	–	2400	4196
Total Input Cost (USD)	5148	3847	5641
a. Catla	2519 kg/ha/yr @ USD 2.17 per kg = USD 5462	2838 kg/ha/yr @ USD 2.75 per kg = USD 7795	2838 kg/ha/yr @ USD 2.75 per kg = USD 7795
b. Rohu	2009 kg/ha/yr @ USD 2.17 per kg = USD 4356.20	2125 kg/ha/yr @ USD2.75 per kg = USD 5835.46	2125 kg/ha/yr @ USD 2.75 per kg = USD 5835.46
c. Mrigal	1511 kg/ha/yr @ USD 2.17 per kg = USD 3276	1643 kg/ha/yr @ USD 2.75 per kg = USD 4512.6	1643 kg/ha/yr @ USD 2.75 per kg = USD 4512.6
d. Vermicompost	–	25,000 kg @ USD 0.065 per kg = USD 1626	60,000 kg @ USD 0.065 per kg = USD 3903
Total income (USD)	13,095	19,770	22,047

**Table 11:** Payback period, net present value and internal rate of return in OCS and CCS.

Culture systems	Profit (USD)	Payback period (years)	Net Present Value (USD)	Internal Rate of Return (%)
Organic culture system(OCS)	32328^a^	1.75^a^	106218.75^a^	51.3^a^
Conventional culture System(CCS)	15894^b^	1.82^a^	51117.03^b^	50.7^a^

^a,b^Means superscripted with different letters in rows are significantly different (*P* < 0.05).

#### Payback period

The payback periods in OCS and CCS culture systems are presented in Table 11. The values of payback period in the CCS and OCS culture systems are 1.82 year and 1.75 year respectively. The differences in payback period in two culture systems, i.e., CCS and OCS are insignificant (*P* > 0.05). It is the period to get back only the initial outlay. It means that the project gives an actual return to the fisher after 2 years both from CCS and OCS out of the expected 10 years of the project life time.

#### Net present value (NPV)

The NPV estimated in the study are presented in Table 11. As the NPV is greater than zero in all the treatments, all of them may be accepted. However, in the financial theory, if there is a choice between two mutually exclusive alternatives, the one yielding the higher NPV should be selected. NPV is as high as USD 106218.75 for OCS and is as low as USD 51117.03 in CCS.

#### Internal rate of return

The investment with a higher IRR is usually the better investment. The IRR values calculated in different treatments of the study are presented in Table 11. The IRR values are found to be more than 50% in all the treatments. The highest value of IRR is achieved in OCS (51.3%) followed by CCS (50.7%). The values of IRR in both are quite high and all of these projects are acceptable.

#### Sensitivity analysis of various inputs

Among the different items in terms of capital as well as recurring inputs, it is an important to identify the items affecting the economic viability of the project significantly. Special attention needs to be paid for economic utilization of those items during the culture operation. The sensitivity analysis was carried out for the said project. The sensitive parameters affecting the economics of the project were identified as cost of soil excavation, input cost, and cost of fingerlings, construction cost of vermicomposting unit, cost of vermicomposting, cost of cultivation of maize and soybean, sale price of organic fishes and sale price of vermicomposting. The variation in the values of NPV and IRR with 20% increase or decrease in the cost of the sensitive parameters is presented in Table 12. The percentage deviation in the values from its original is also estimated for comparison. It can be observed from the above table that except total fish production and sale price of the organic fishes, all other parameters are less sensitive as their variation from their original values are estimated to be less than 5% in terms of NPV and IRR. NPV is much more reliable when compared to IRR and is the best approach when ranking projects that are mutually exclusive. The sale prices of organic fishes are found to be the most sensitive parameters as they increase or decrease the NPV and IRR significantly with 20% increase or decrease of the sale price. With 20% increase in the sale price of organic fishes, the NPV and IRR increase by 28.2% and 7.31% respectively, whereas decreasing those quantities by 20%, the NPV and IRR decrease by 28.2% and 9.5% respectively.

**Table 12:** Sensitivity analysis of different items for OCS.

Item	Economic parameter				
	Particulars	NPV (USD)	% Increase or decrease	IRR (%)	% Increase or decrease
	Actual value	106,219	–	51.28	–
Cost of soil excavation	20% (+)	105,496	0.70% (−)	50.81	0.92% (−)
	20% (−)	106,942	0.70% (+)	51.76	0.94% (+)
Input cost	20% (+)	97,225	8.50% (−)	50.03	2.44% (−)
	20% (−)	115,212	8.50% (+)	52.43	2.24% (+)
Construction cost of vermicompost unit	20% (+)	102,798	3.20% (−)	49.13	4.20% (−)
	20% (−)	109,640	3.20% (+)	53.65	4.62% (+)
Cost of vermicomposting	20% (+)	99,611	6.20% (−)	50.39	1.74% (−)
	20% (−)	112,827	6.20% (+)	52.11	1.60% (+)
Cost of fingerlings	20% (+)	105,742	0.45% (−)	51.21	0.136% (−)
	20% (−)	106,696	0.45% (+)	51.35	0.136% (+)
Cost of cultivation of maize and soybean	20% (+)	105,180	0.98% (−)	51.13	0.29% (−)
	20% (−)	107,257	0.98% (+)	51.43	0.29% (+)
Sale price of organic fishes	20% (+)	136,160	28.20% (+)	55.03	7.31% (+)
	20% (−)	76,277	28.20% (−)	46.41	9.50% (−)
Sale price of vermicompost	20% (+)	112,259	5.70% (+)	52.02	1.44% (+)
	20% (−)	100,179	5.70% (−)	50.50	1.52% (−)

The sensitivity analysis of the various parameters clearly shows that profitability of the fish farming investment is most sensitive to the sale price of organic fishes. However, in case of CCS systems, apart from sale price of fishes, feed cost also significantly affects the financial status of the farming project^50^.

In this organic fish culture system, the main advantage is that a part of the vermicomposting is directly used as fertilizer and feed in the culture of fishes and the remaining part can be sold in the market for further income generation.

## Conclusions

The economic feasibility of organic aquaculture of Indian major carp culture was evaluated through the study. Based on the results of economic feasibility study in the present work, the following specific conclusions are drawn:

- Highest production to the tune of 19 tons of Indian major carps per ha is obtained in organic culture system.
- In addition to fish yield, production of vermicompost is an additional benefit for the organic culture system.
- Highest net present value of USD 106218.75, a payback period of about two years and an IRR of 51% are achievable for organic culture system assuming the project period to be 10 years.
- Production of fish, vermicomposting and with organic culture system is proved to be the best available technique.
- Profitability of the organic fish farming investment is most sensitive to the sale price of the organic fishes.

On the basis of the study, organic culture practice for Indian major carp is strongly recommended for long term benefit in terms of quality product, human health and protection of environment.

## Data Availability

The datasets generated during and/or analyzed during the current study are available from the corresponding author upon reasonable request.
